# Draft Genome Sequence of the First Isolate of Extensively Drug-Resistant (XDR) *Mycobacterium tuberculosis* in Malaysia

**DOI:** 10.1128/genomeA.00056-12

**Published:** 2013-01-24

**Authors:** Kee Peng Ng, Su Mei Yew, Chai Ling Chan, Jennifer Chong, Soo Nee Tang, Tuck Soon Soo-Hoo, Shiang Ling Na, Hamimah Hassan, Yun Fong Ngeow, Chee Choong Hoh, Kok Wei Lee, Wai Yan Yee

**Affiliations:** a Department of Medical Microbiology, Faculty of Medicine, University of Malaya, Kuala Lumpur, Malaysia; b Codon Genomics SB, Jalan Bandar Lapan Belas, Pusat Bandar Puchong, Selangor Darul Ehsan, Malaysia

## Abstract

The emergence of the global threat of extensively drug-resistant (XDR) *Mycobacterium tuberculosis* reveals weaknesses in tuberculosis management and diagnostic services. We report the draft genome sequence of the first extensively drug-resistant *Mycobacterium tuberculosis* strain isolated in Malaysia. The sequence was also compared against a reference strain to elucidate the polymorphism that is related to their extensive resistance.

## GENOME ANNOUNCEMENT

The emergence of multidrug-resistant (MDR) and extensively drug-resistant (XDR) *Mycobacterium tuberculosis* is a major public health problem that threatens tuberculosis (TB) care and control in many countries (1). XDR *Mycobacterium tuberculosis* strain UM 1072388579 was isolated from a 57-year-old man. The isolate was resistant to amikacin, capreomycin, ciprofloxacin, ethionamide, isoniazid, kanamycin, ofloxacin, rifampin, and streptomycin but sensitive to ethambutol and para-salicylic acid.

The genomic DNA of UM 1072388579 was sequenced to 240× coverage with a 500-bp insert size library strategy, which consisted of 5,861,112 paired-end reads, using the Illumina HiSeq 2000 sequencer machine. After preprocessing, 2,800,000 high-quality paired-end reads (100-fold depth) were subsampled and assembled using Velvet v1.1.07 (2), producing 213 contigs (≥200 bp). The contigs were then scaffolded and gap filled with SSPACE v2.0 (3) and GapFiller v1.10 (4) programs by utilizing all paired-end information available. Final assembly consisted of 89 scaffolds (≥1,000 bp) with 158 contigs comprising a total length of 4.3 Mb. The assembly has an N50 scaffold size of 108,779 bp and *G+C* content of 65.4%. Protein coding sequences, rRNAs, and tRNAs of the XDR *M. tuberculosis* genome were predicted using GeneMarkS (5), RNAmmer 1.2 server (6), and tRNAscan-SE v1.23 (7), respectively. Our pipeline predicted 4,099 coding DNA sequences (CDS) with length ≥33 amino acids (aa), 45 tRNAs, and 3 rRNAs (5S, 16S, and 23S). Annotation of the coding sequences predicted was performed by a BLAST search against the NCBI nonredundant database with 4,069 (99.3%) genes annotated with function. All annotated genes were subjected to Gene Ontology classification and Kyoto Encyclopedia of Genes and Genomes (KEGG) pathways analysis.

The genome sequence is about 93% complete compared to the F11 reference genome, which has a complete genome size of 4.42 Mb. A polymorphism study of the XDR *M. tuberculosis* genome was carried out by comparative analysis against the F11 genome (8) to identify the difference between intergenic and coding regions of drug-susceptible versus extensively drug-resistant strains. A total of 1,455 polymorphisms were observed in our comparative study, with 1,196 of these located in the coding regions of the genome. An insertion at the *tlyA* gene (a determinant for extensively drug-resistant *M. tuberculosis*) conferring capreomycin resistance to *M. tuberculosis* was observed. Mutations of other drug resistance sites such as *ethA* (ethionamide), *gidB* (streptomycin), *gyrA* and *gyrB* (fluoroquinolones), *pncA* (pyrazinamide), *katG* and *ndh* (isoniazid), and *rpoB* (rifampin) were also observed. The draft sequence of the XDR *M. tuberculosis* UM 1072388579 genome provides a genome sequence that may lead to better understanding of the genetic molecular differences in acquired drug resistance of XDR *M. tuberculosis* isolates locally and in other parts of the world.

### Nucleotide sequence accession number.

The nucleotide sequences of the *M. tuberculosis* UM 1072388579 genome have been deposited in DDBJ/EMBL/GenBank under accession number AMXW00000000.
